# Chimeric Sex-Determining Chromosomal Regions and Dysregulation of Cell-Type Identity in a Sterile *Zygosaccharomyces* Allodiploid Yeast

**DOI:** 10.1371/journal.pone.0152558

**Published:** 2016-04-11

**Authors:** Melissa Bizzarri, Paolo Giudici, Stefano Cassanelli, Lisa Solieri

**Affiliations:** Department of Life Sciences, University of Modena and Reggio Emilia, Via Amendola 2, 42122, Reggio Emilia, Italy; Institut de Genetique et Microbiologie, FRANCE

## Abstract

Allodiploidization is a fundamental yet evolutionarily poorly characterized event, which impacts genome evolution and heredity, controlling organismal development and polyploid cell-types. In this study, we investigated the sex determination system in the allodiploid and sterile ATCC 42981 yeast, a member of the *Zygosaccharomyces rouxii* species complex, and used it to study how a chimeric mating-type gene repertoire contributes to hybrid reproductive isolation. We found that ATCC 42981 has 7 *MAT*-like (*MTL*) loci, 3 of which encode α-idiomorph and 4 encode **a**-idiomorph. Two phylogenetically divergent *MAT* expression loci were identified on different chromosomes, accounting for a hybrid **a**/α genotype. Furthermore, extra **a**-idimorph-encoding loci (termed *MTL***a** copies 1 to 3) were recognized, which shared the same *MAT***a**1 ORFs but diverged for *MAT***a**2 genes. Each *MAT* expression locus was linked to a *HML* silent cassette, while the corresponding *HMR* loci were located on another chromosome. Two putative parental sex chromosome pairs contributed to this unusual genomic architecture: one came from an as-yet-undescribed taxon, which has the NCYC 3042 strain as a unique representative, while the other did not match any *MAT*-*HML* and *HMR* organizations previously described in *Z*. *rouxii* species. This chimeric rearrangement produces two copies of the *HO* gene, which encode for putatively functional endonucleases essential for mating-type switching. Although both **a** and α coding sequences, which are required to obtain a functional cell-type **a**1-α2 regulator, were present in the allodiploid ATCC 42981 genome, the transcriptional circuit, which regulates entry into meiosis in response to meiosis-inducing salt stress, appeared to be turned off. Furthermore, haploid and α-specific genes, such as *MAT*α1 and *HO*, were observed to be actively transcribed and up-regulated under hypersaline stress. Overall, these evidences demonstrate that ATCC 42981 is unable to repress haploid α-specific genes and to activate meiosis in response to stress. We argue that sequence divergence within the chimeric **a**1-α2 heterodimer could be involved in the generation of negative epistasis, contributing to the allodiploid sterility and the dysregulation of cell identity.

## Introduction

Ploidy variation has played a major role in the evolution of many extant eukaryotic lineages [1]. In yeasts, numerous studies have demonstrated how variations in the ploidy state took place frequently during evolutionary history [2] [3]. Allodiploid offspring often have strong selective disadvantages due to their sterility [4]. However, in some instances, the increased genome size and complexity of allodiploids may enhance heterosis and/or adaptive flexibility [5], particularly at the edges of the ancestral species' range, where they are more likely to encounter stress [6] [7]; [8]. Life history models note that there is an intricate interplay between ploidy variation and alterations of mating, meiosis and sporulation patterns [9]. Consequently, the ploidy state affects the genetic composition at sex-determining loci, giving rise to the ploidy-dependent initiation of dedicated transcriptional programs [10].

In the well-studied model organism *Saccharomyces cerevisiae*, the meiosis of diploid cells is triggered by environmental cues, such as nutrient depletion, and gives rise to four haploid meiotic spores with two distinct mating-types (*MAT***a** and *MAT*α). Haploid spores eventually re-establish diploid *MAT***a**/*MAT*α lines by one of three processes: (1) by mating with their own mitotic daughter cells after switching their mating-type in a process catalysed by the endonuclease *HO* (HOmothallism); (2) by mating with another spore created by the same meiotic event (intratetrad mating); or, more rarely, (3) by mating with an unrelated individual (outcrossing) [11]. The **a**1-α2 protein heterodimer is one of the master regulators of cell identity and sexual development (as reviewed in [12] and [13], respectively). The **a**1 and α2 proteins are homeodomain transcriptional factors encoded by the *MAT***a**1 and *MAT*α2 genes at the active *MAT***a** and *MAT*α loci, respectively. In *MAT***a**/*MAT*α diploid cells, α2 interacts with **a**1 to bind DNA as a heterodimer and transcriptionally repress mating genes, preventing polyploidy and aneuploidy. Furthermore, the **a**1-α2 heterodimer inhibits the expression of haploid-specific genes (h-sgs) and activates the developmental switch from mitosis to meiosis under appropriate stress stimuli [14] [15]. Two h-sg targets of negative regulation by the **a**1-α2 heterodimer are *MAT*α1 (encoding the transcription factor that positively regulates the α-specific genes) and *HO* genes (encoding an endonuclease, which cleaves a specific DNA sequence at the *MAT* locus during the first step of mating-type switching) [16]. Furthermore, **a**1-α2 heterodimer controls the sexual development by inhibiting the *RME1* (*Repressor of Meiosis* 1) gene. In haploid cells, the RME1 transcriptional factor prevents the expression of the *IME1* (*Inducer of Meiosis* 1) gene [17] [18] and positively regulates the transcription of adhesion-specific genes, such as the *FLO11* gene, in response to nutrient depletion [19]. In diploid cells, the **a**1-α2 heterodimer binds to the *RME1* gene promoter, repressing its transcription and thereby relieving *IME1* gene repression. The **a**1-α2 repressor complex directly controls entry into meiosis through the regulation of the *IME4* gene, which is required for the full expression of *IME1* [20]. The **a**1-α2 heterodimer negatively regulates the antisense (AS) long non-coding RNA (lncRNA) [21] [22]. This lncRNA (also termed as *Regulator of Meiosis* 2 or *RME2*) is transcribed in haploid cells and blocks the expression of the *IME4* gene. In diploid cells, the **a**1-α2 complex represses *RME2* expression, allowing sense (S)-*IME4* to be transcribed under starvation conditions.

Yeasts of the non-whole genome duplication (non-WGD) *Zygosaccharomyces rouxii* species complex are adapted to grow in food with high solute concentrations and frequently experience variations in ploidy, resulting in different modes of reproduction [23]. This complex includes the haploid heterothallic or homothallic (self-fertile) *Z*. *rouxii* species, which undergoes mating and subsequent meiosis under salt stimuli [23] [24]; the diploid *Zygosaccharomyces sapae* species, which reproduces mainly by clonality and ascospores are rarely observed [25]; and a group of anueploid/allodiploid strains of unclear taxonomical position (termed mosaic lineage) [23] [26]. Within the latter, the allodiploid ATCC 42981 strain has been extensively studied for its ability to withstand high concentrations of alkali metal cations and for its capability to produce glycerol under salt stress [27] [28]. From a molecular point of view, the ATCC 42981 genome displays rDNA heterogeneity [26] [29], additional chromosomes compared to *Z*. *rouxii* [28], and diploid DNA [26]. Gordon and Wolfe [29] showed that the ATCC 42981 genome contains two partially divergent complements, namely the T- and P- subgenomes, which could arise from a recent allodiploidization event between *Z*. *rouxii* carrying the T- subgenome and another not-yet-recognized species harbouring the P- subgenome (so far represented by the unique strain NCYC 3042 and referred to as *Zygosaccharomyces pseudorouxii* nom. inval. by James et al. [30]). Despite having a DNA diploid content, the ATCC 42981 strain has not been observed to undergo meiosis under different standard and stress growth conditions [25].

Because gene information retained at the *MAT* expression loci is essential to ensure appropriate haploid/diploid cell-type identity and functional cell development, several efforts have recently been made to characterize *MAT* loci in haploid *Z*. *rouxii* [31] and diploid *Z*. *sapae* [32], but not in the allodiploid ATCC 42981 strain. Like *S*. *cerevisiae*, haploid *Z*. *rouxii* wild strains possess a three-cassette system consisting of *MAT***a** or *MAT*α expression loci and two silent cassettes of both idiomorphs, *HMR* and *HML*, respectively, which act as donor sequences during the mating-type switching presumably catalysed by HO endonuclease [33] [34]. *S*. *cerevisiae* maintains both *HML* and *HMR* cassettes at different locations on the same chromosome harbouring the *MAT* expression locus, whereas *Z*. *rouxii* only conserves the *MAT*/*HML* linkage on chromosome C and the *HMR* locus on chromosome F [34]. This structural organization implies that, differently from *S*. *cerevisiae*, *Z*. *rouxii* exploits the ectopic recombination between non-homologous chromosomes to switch the mating-type. Congruently, Watanabe et al. [31] found that sex chromosomes represent hyper-mutational hotspots, with flanking regions of *MAT*, *HML* and *HMR* cassettes remaining highly variable among *Z*. *rouxii* haploid strains. Compared to *Z*. *rouxii*, the *Z*. *sapae* diploid species has one more copy of the *HO* gene and displays an unusual **a**ααα genotype, with a redundant number of divergent *MAT*α loci but without any *HMR* silent locus. This complex architecture of sex-determining chromosomal regions prevents mating-type switching due to the lack of the *HMR* cassette and hampers sexual development due to the imbalance in divergent mating-type genes.

Here, we determined the structure and functions of the three-locus sex-determining system in the allodiploid ATCC 42981 strain. The transcriptional profiling of these genes under meiotic-inducing salt stress revealed how the hybrid genetic configuration at the *MAT* loci contributes to allodiploid sterility. To the best of our knowledge, our report presents the first evidence that a chimeric sex-determination system accounts for the incomplete silencing of the h-sg program and contributes to the prevention of switching from mitosis to meiosis in allodiploid yeasts.

## Materials and Methods

### Strains, culture conditions and mating test

The *Zygosaccharomyces* strains used in this work are listed in Table 1. Strains were routinely cultured at 28°C in YPD (1% w/v yeast extract, 1% w/v peptone, 2% w/v dextrose) medium with or without 15% (w/v) agar and stored at 4°C. For long-term preservation, strains were stored at -80°C in YPD medium containing 25% glycerol (v/v) as a cryopreservative. To increase the probability of observing conjugated or un-conjugated spore-containing ascii, sporulation was tested by inoculating the early stationary phase culture of ATCC 42981 on five different media [YPD, YPDA, malt extract agar (MEA; Difco), MEA supplemented with 6% (w/v) NaCl (6%NaCl-MEA), and YNB5%GNaCl (1% w/v yeast extract, 5% w/v dextrose, 6.7 g/l yeast nitrogen base, 2.0 M NaCl)] for 3 weeks.

**Table 1:** Details of strains used in the present study. Ploidy data were obtained from Solieri et al. [23] [2,6], while the genotype of strain ABT301^T^ at the active *MAT* loci was obtained from Solieri et al. [32].

Strains	Other collections	Source	Current taxonomical positions	Mating-type/thallism	Spore	Ploidy ratio
CBS 732^T^	NCYC 568, NRRL Y-229	Grape must	*Z*. *rouxii*	*MAT*α*/*homothallic	-	1.3
CBS 4837	NYC 1682, NRRL Y2547	Miso	mosaic lineage	*MAT***a**/heterothallic	+	1.96
CBS 4838	NRRL Y2584	Miso	mosaic lineage	*MAT*α/heterothallic	+	1.90
ATCC42981	-	Miso	mosaic lineage	nd	nd	2.1
ABT301^T^	CBS 12607, MUCL54092	TBV	*Z*. *sapae*	**a**ααα*MAT*α/*MAT*α/heterothallic	+	2.0
NCYC 3042	CBS 9951	Soft drink	*Z*. *pseudorouxii*nom. inval.	nd		nd

nd, not determined; TBV, Traditional Balsamic Vinegar.

To study sexual compatibility, 2-to-4-day-old cultures of the ATCC 42981 strain were incubated alone or in a mixture with mating-tester strains *Z*. *rouxii* CBS 4837 (mating-type **a**) or CBS 4838 (mating-type α) in both MEA and 6%NaCl-MEA media at 27°C for 3 weeks. Samples were examined microscopically every week using phase-contrast optics to detect conjugation.

The YNB5%G [1% w/v yeast extract, 5% w/v dextrose, 6.7 g/l yeast nitrogen base (Difco)] and the YNB5%GNaCl (1% w/v yeast extract, 5% w/v dextrose, 6.7 g/l yeast nitrogen base, 2.0 M NaCl) media were used for RNA extraction from cells grown under unstressed and salt-stressed conditions, respectively.

### PCR conditions and sequencing

Genomic DNA (gDNA) extraction was performed by a phenol-based method from stationary grown cells after mechanical lysis according to Hoffman and Winston [35]. The quantity of DNA was evaluated spectrophotometrically using a NanoDrop ND-1000 device (Thermo Scientific). All PCR reactions were performed on a T100 Thermalcycler (Bio-Rad) in a 25-μl reaction volume containing 200 ng of template gDNA. For PCR amplification < 2 kb rTAQ DNA polymerase (Takara, Japan) and for PCR amplification ≥ 2 kb Phusion Hot Start II Polymerase (Thermo Scientific, Waltham, MA) along with buffer HF (5x) or LA Taq DNA polymerase (Takara, Japan) with GC buffer I (2x), were used according to the manufacturer’s instructions. All primers used in this study are listed in S1 Table; primers were designed with Primer 3 software (http://primer3.sourceforge.net/) and provided by either MWG (Heidelgerg, Germany) or BMR Genomics (Padova, Italy). The PCR products were resolved on 1.2% (w/v) agarose gels stained with ethidium bromide and their size was estimated by comparison with 100 bp or 1 kb DNA Ladder Plus (Fermentas) as molecular size markers. PCR products were purified using the DNA Clean & Concentrator^™^-5 (DCC^™^-5) Kit (Zymo Research) according to the manufacturer’s instructions. Moreover, when required, DNA fragments were purified from 1% agarose gels using the Gene JET Gel Extraction Kit (Thermo Scientific, Waltham, MA). Finally, all PCR products were sequenced on both strands through a DNA Sanger sequencing process performed by either MWG (Heidelgerg, Germany) or BMR Genomics (Padova, Italy).

### Genomic walking procedure

The overall strategy for determining mating-type-like (*MTL*) loci is depicted in S1 Fig (panel A). Briefly, three *MAT*α copy-specific primer pairs were designed on the *ZsMTL*α copies 1, 2 and 3 cassettes previously cloned in *Z*. *sapae* and used to clone the corresponding *MAT*α1 (partial) and *MAT*α2 (full-length) coding regions in the ATCC 42981 genome (S1 Fig and S1 Table). Complete *MAT*α1 loci were obtained in a second round of PCR walking using reverse primers (A, B, C and A_D) built on the 3’-end regions potentially flanking the *MAT* and *HML*/*HMR* cassettes [31] [32] (S1 Fig). Similarly, three primer pairs were used to amplify putative *MTL***a** loci in the ATCC 42981 strain (S1 Fig, panel B). Two primer pairs were designed to specifically amplify the *ZsMAT***a**1 and *ZsMAT***a**2 genes, respectively, whereas a third primer pair was built to completely include *MAT***a**1 ORF and partially *MAT***a**2 ORFs, respectively. In order to further extend the *MAT***a**2 coding DNA sequence, a second round of PCR walking was performed by combining a *MAT***a**2-specific reverse primer and all the available forward primers (1, 2 and 3) built on the 5’-end regions flanking the *MAT* and *HML*/*HMR* cassettes [31] (S1 Fig). The DNA regions flanking *MTL***a** and *MTL*α loci were characterized through the semi-nested and direct PCR approaches as previously reported [32] (S2 Fig).

The overview of the strategy employed for ATCC 42981 *HO* gene characterization is summarized in S3 Fig, and a detailed primer list is given in S1 Table.

### Bioinformatics analyses

Sequences were assembled and edited using DNAStar Software (DNASTAR, Inc. Madison, Wisconsin USA). Multiple nucleotide and amino acid sequence alignments were performed using Clustal W2 [36]. Searches for nucleotide and protein sequence homologs were carried out in the GenBank database with Blastn and Blastp algorithms, respectively [37]. Phylogenetic analysis was conducted on aa sequences using MEGA6 [38]. The phylogenetic relationship was inferred using the neighbour-joining (NJ) method. Support percentages for the nodes of NJ-trees were computed using bootstrapping analysis with 1,000 replications and were shown next to the branches when ≥ 60%. For domain identification, Pfam-searches [39] were run on http://pfam.sanger.ac.uk/. Structure predictions were obtained with Jpred3 [40] and validated according to Martin et al. [41]. Sequences were submitted to the EMBL/GenBank databases under the accession numbers from KT598024 to KT598027 and from KT694298 to KT6942302.

### PFGE-Southern blotting assays

Chromosomal DNA preparation in plug, pulsed-field gel electrophoresis (PFGE), and Southern blotting assays were performed as previously reported [26] [32]. Primers engaged in probe synthesis for Southern blot analysis are listed in S1 Table.

### Subgenome assignment of mating-type and *HO* gene copies

PCR subgenome genotyping was performed using gDNA extracted from Z. *pseudorouxii* NCYC 3042 as a T- subgenome template. PCR amplification of all *MAT*α1, *MAT*α2, *MAT***a**1, *MAT***a**2, and *HO* gene copies present in ATCC 42981 genome was carried out with the primer pairs listed in S1 Table. To avoid false negative results, we tested two alternative primer pairs for each target gene. ATCC 42981 gDNA was also included as a positive control. *Z*. *rouxii* CBS 732^T^ gDNA was not included in PCR reactions as one of the two putative parental genetic complements because its genome project was available.

### RNA extraction and RT-PCR

*Zygosaccharomyces* cells were pre-cultured in YPD medium for 24 h at 28°C under shaking conditions (150 rpm), washed in physiological solution (9 g/l NaCl), and used to inoculate two sets of baffled Erlenmeyer flasks (E-flasks) containing 70 ml of YNB5%G (standard growth condition) and YNB5%GNaCl (hyperosmotic growth condition) media, respectively (initial OD_600nm_ 0.02–0.04). Inoculated E-flasks were incubated at 28°C under shaking conditions (150 rpm) and yeast growth was spectrophotometrically monitored at 600 nm two times a day. Cells at the stationary phase (no change in OD measurement detected in at least three consecutive readings) were harvested and frozen at -80°C. RNA extractions were carried out using the ZR Fungal/Bacterial RNA MicroPrep^™^ Kit (ZymoReasearch, Irvine, California) according to the manufacturer’s instructions. Purified RNAs were submitted to additional DNase digestion in solution and cleaned up with the RNA Clean & Concentrator^™^-5 Kit (ZymoReasearch, Irvine, California).

Total RNAs were reverse transcribed using 0.5 μM oligo (dT) and RevertAid H Minus Reverse Transcriptase (Thermo Scientific, Waltham, USA) according to the manufacturer's instructions. In the case of *IME4* transcript analyses, instead of oligo (dT) the forward and reverse primers ZrIME4F1 and ZrIME4R1 were used for the cDNA synthesis of AS-*IME4* lncRNA and S-*IME4* mRNA, respectively (S1 Table). Then a common pair of internal primers (ZrIME4_F2/ZrIME4_R2; S1 Table) was used to amplify the same cDNA fragment from each template, if any. Experiments were carried out with three biological replicates and non-reverse transcribed (NRT) controls were performed for each biological replicate. cDNA template (25 ng) was PCR-amplified with primers listed in S1 Table and successful amplification was checked by electrophoresis in 2% agarose gel in 0.5X TBE Buffer.

### Quantitative PCR (qPCR) assays

For relative expression level analysis, qPCR reactions were performed using 1 ng/μl of cDNA, 0.3 μM of each primer, and the Maxima^™^ SYBR Green/ROX qPCR Master Mix (Fermentas, USA) according to the manufacturer’s instructions. Primers were designed to selectively amplify *MAT***a**1 exons as well as different copies of *MAT*α1, *MAT*α2 and *HO* genes (S1 Table). PCR efficiency was *in silico* predicted for each primer set using the open source tool Primer Efficiency (http://srvgen.upct.es/index.html; [42]). Preliminary, the presence and the estimated size of amplicons were checked by RT-PCR on cDNA from stationary-phase cells (three biological replicates) under standard conditions, whereas unspecific amplifications and primer-dimer formation were checked by melting curve analysis after RT-qPCR assay. All qPCR reactions were run in the Applied Biosystems 7300 Real-Time PCR instrument (Applied Biosystem, Foster City, CA, USA). The *Z*. *rouxii* housekeeping gene *ACT1* (Zr*ACT1*; XM002497273) was used as a reference gene, according to Leandro et al. [43]. The relative expression of different gene transcripts was calculated by the ΔΔCt method and converted to the relative expression ratio (2−ΔΔCt) for statistical analysis [44]. For this purpose, a dilution series of standard points from pooled control cDNAs was exploited (concentration range 10–0.02 ng/μl) in technical triplicates. Amplification profiles, baselines and thresholds were analysed with 7300 SDS 1.4. PCR-Miner [45] [46] was applied as an alternative method when the low gene expression level did not provide enough dynamic range to build a reliable standard curve. In this case, reaction efficiency and the fractional cycle number at threshold (Ct) were estimated by relying on the kinetics of individual PCR reactions containing 5 ng of cDNA template from three biological replicates (salt-treated and controls), including six technical replicates each.

## Results

### Morphological characterization and mating test

We first assessed the mating behaviour of the ATCC 42981 strain in pure and mixed cultures with the *Z*. *rouxii* mating partners CBS 4837 (mating-type **a**) and CBS 4838 (mating-type α), respectively. Examination under the microscope did not show any evidence of mating reaction of ATCC 42981 cells neither with *Z*. *rouxii* CBS 4837 or CBS 4838 tester strains, even after 3 weeks of incubation both on MEA and 6% NaCl-MEA media (data not shown). Even if rare mating events cannot be ruled out, this result suggests that the ATCC 42981 strain did not respond to *Z*. *rouxii* pheromone signalling or that there was an imbalanced or deficient organization in *MTL* loci. Based on our previous observations [25], ATCC 42981 cells grown on MEA medium displayed an adhesive phenotype with clamps on mother and daughter cells that remained attached to each other, but they were not able to form ascii in pure culture (data not shown). In contrast, *Z*. *sapae* ABT 301^T^ rarely forms ascii at the same conditions [25]. As salt has been reported to induce meiosis in *Zygosaccharomyces* yeasts [24], we tested the capability of the ATCC 42981 strain to form ascospores in YNB5%G NaCl and 6%NaCl-MEA media. No conjugated ascii were observed over time in any media tested. Furthermore, an increase in the adhesive phenotype was detected in cells grown under salt stress (data not shown).

### Isolation and characterization of *MTL*α loci in ATCC 42981

Three *MTL*α loci, termed *ZsMTL*α copies 1 to 3, have been previously described in *Z*. *sapae* as regions containing phylogenetically distinct α1 and α2 genes, respectively [32]. To establish whether strain ATCC 42981 also displays a similar genetic configuration, we exploited a PCR walking strategy. This assay relies on *MAT*α copy-specific primers designed on the corresponding *ZsMTL*α cassettes [32]. PCR reactions were positive using *MTL*α copies 1 and 2-specific primers, whereas negative results were scored for any *MTL*α copy 3-specific primer set tested. We sequenced two distinct *MTL*α loci (hereafter referred to as *MTL*α copies 1 and 2) in ATCC 42981. Both loci consisted of two *MAT* genes encoding α1 and α2 proteins placed in opposite directions and separated by an intervening region of 343 bp. The *MAT*α1 genes from *MTL*α loci 1 and 2 (termed *MAT*α1 copies 1 and 2, respectively) were divergent from each other for 17 nt substitutions, whereas the *MAT*α2 from *MTL*α loci 1 and 2 (termed *MAT*α2 copies 1 and 2, respectively) displayed 16 nt substitutions. The deduced proteins MATα1 and MATα2 copies 1 were 87.00% and 78.67% identical to the deduced protein MATα1 and MATα2 copies 2, respectively. BLASTp search against the NCBI database showed that proteins MATα1 and MATα2 copies 1 are more similar to *Z*. *rouxii* orthologs (termed ZrMATα1 and ZrMATα2) than the paralogous proteins MATα1 and MATα2 copies 2 in the ATCC 42981 genome.

**Fig 1:**
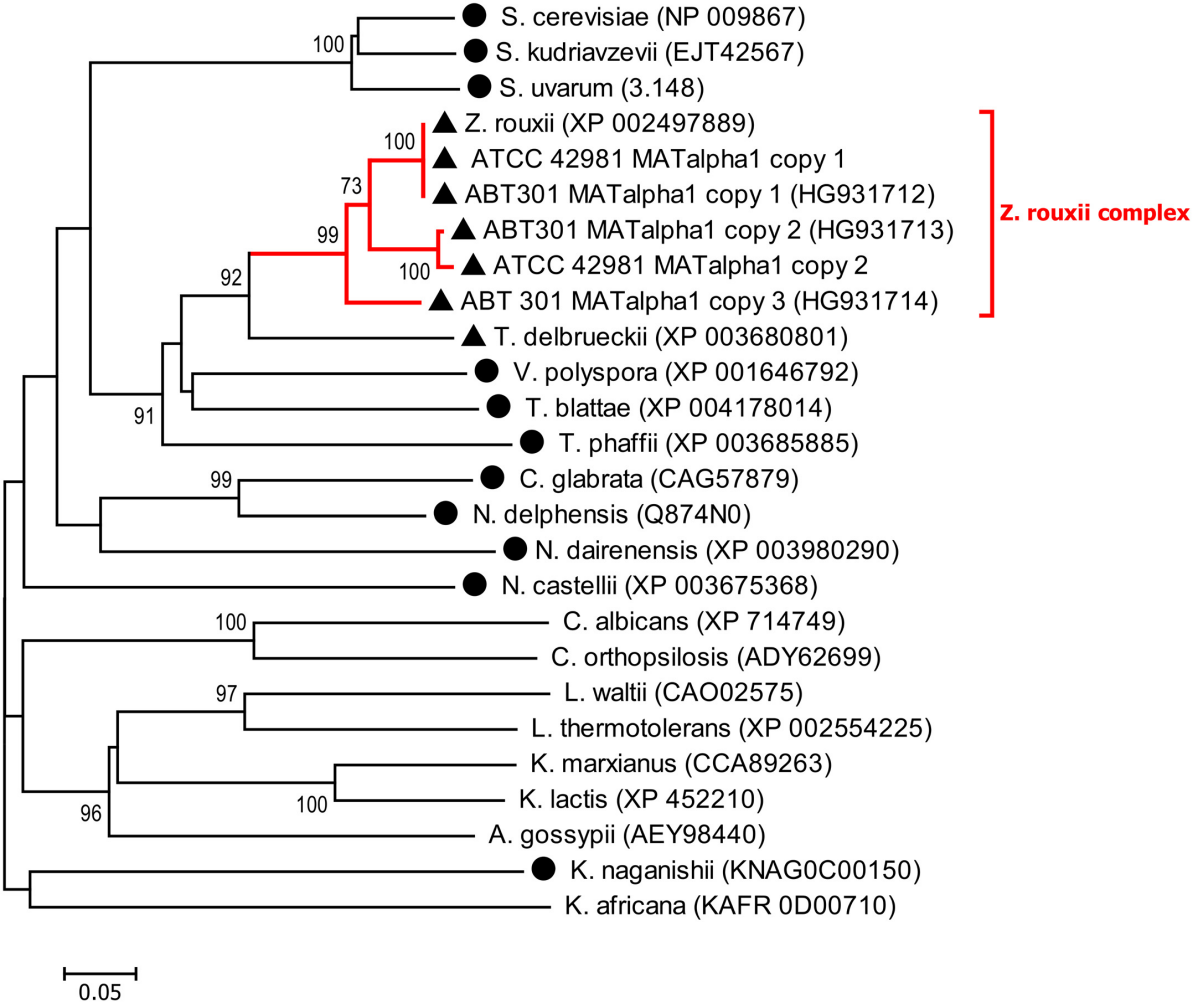
Phylogenetic analysis of MATα1 proteins. The neighbour-joining (NJ) tree shows the phylogenetic relationships between the allodiploid strain ATCC 42981 and other hemiascomycetes inferred from MATα1 proteins. Numbers on branches indicate bootstrap support percentages (1,000 pseudoreplicates) higher than 60% from NJ. The red branch indicates the *Z*. *rouxii* yeast complex, which includes *Z*. *rouxii* MATα1, ATCC 42981 MATα1 copies 1 and 2, *Z*. *sapae* MATα1 copies 1, 2, and 3 sequences. The dark dot indicates WGD species, whereas the dark triangle indicates non-WGD species with the *HO* gene.

The NJ-based phylogenetic analysis was carried on MATα1 sequences from representative WGD and non-WGD species. As expected, ATCC 42981 MATα1 copy 2 protein did not group to *Z*. *rouxii* MATα1, but was instead clustered separately (bootstrapping value of 99%) together with ZsMATα1 copy 2 (Fig 1). The alignment of ATCC 42981 MATα1 copies with *Z*. *rouxii* and *S*. *cerevisiae* MATα1 proteins revealed a region of high similarity inside the MATα-HMG domain [41] with three predicted conserved α helices (data not shown).

**Fig 2:**
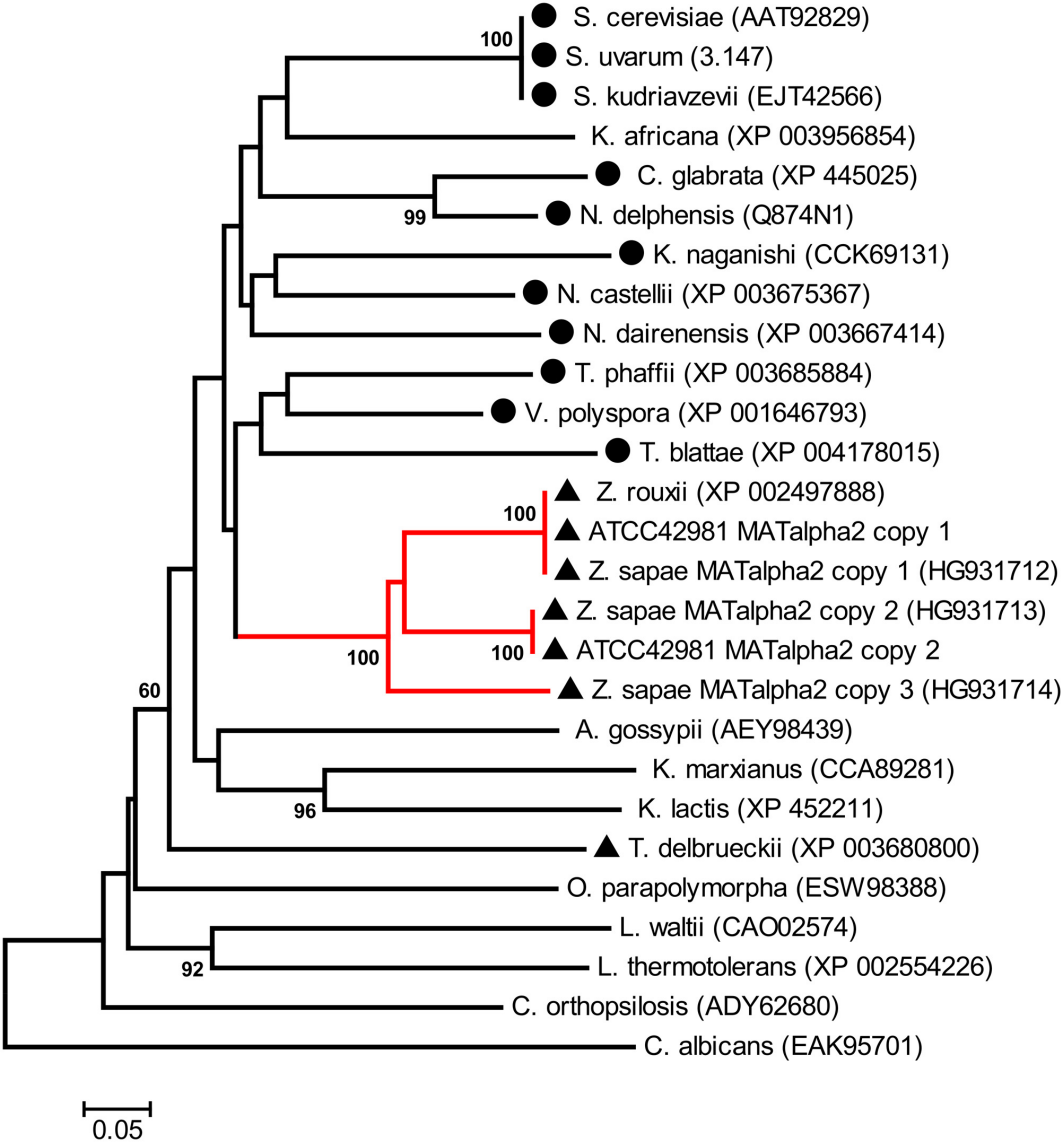
Phylogenetic analysis of MATα2 proteins. The neighbour-joining (NJ) tree shows the phylogenetic relationships between the allodiploid strain ATCC 42981 and other hemiascomycetes inferred from MATα2 proteins. Numbers on branches indicate bootstrap support percentages (1,000 pseudoreplicates) higher than 60% from NJ. The red branch indicates *Z*. *rouxii* complex, which includes *Z*. *rouxii* MATα2, ATCC 42981 MATα2 copies 1 and 2, and *Z*. *sapae* MATα2 copies 1 to 3 sequences. The dark dot indicates WGD species, whereas the dark triangle indicates non-WGD species with the *HO* gene.

Phylogeny inferred from the MATα2 aa sequences of WGD and non-WGD species showed a tree topology congruent with the species relationships established using MATα1 sequences. The ATCC 42981 genome harbours two MATα2 variants that are related but phylogenetically distinct because of the high level of amino acid divergence (Fig 2). In particular, ATCC 42981 MATα2 copy 1 clustered with *Z*. *rouxii* MATα2 (bootstrapping 100%), whereas MATα2 copy 2 was strictly related to *Z*. *sapae* MATα2 copy 2 (bootstrapping 100%). Both copies contained a conserved HD1 class homeodomain, consisting of a three-helix globular domain which contacts both major groove bases and the DNA backbone [47] [48] (data not shown). However, portions of the protein outside the homeodomain, which mediate interactions with accessory proteins, had a different degree of conservation.

### Isolation and characterization of *MTL*a loci in ATCC 42981

In *Z*. *sapae* the **a**-idiomorph encoding *MTL* locus harbours a *MAT***a**1-coding ORF (*ZsMAT***a**1) identical to the *Z*. *rouxii* orthologue (*ZrMAT***a**1) and a *MAT***a**2-coding ORF (*ZsMAT***a**2), which showed a 26-bp deletion compared to *ZrMAT***a**2, resulting in a 9-amino acid shorter protein [32]. To identify *MTL***a** loci in the ATCC 42981 genome, a PCR strategy was used based on primers designed on the *MAT***a**1 and *MAT***a**2 gene sequences in *Z*. *rouxii* and *Z*. *sapae*. We found three *MTL***a** loci, each harbouring *MAT***a**1 and *MAT***a**2 genes in opposite directions separated by a 279-bp long intergenic sequence, which differed for a single SNP (T/G) compared to the corresponding region in *Z*. *sapae*.

Sequence alignments and BLAST-type search revealed that all ATCC 42981 *MTL***a** loci displayed identical *MAT***a**1 genes (100% identity to *ZrMAT***a**1 and *ZsMAT***a**1), whereas differed from each other in the *MAT***a**2-coding ORFs. One *MAT***a**2-coding gene (referred to as the *MAT***a**2 copy 1) was 100% identical to *Z*. *rouxii* counterpart. Another *MAT***a**2 gene (termed the *MAT***a**2 copy 2) encoded an **a**2 protein, which diverged from ZrMAT**a**2 mainly at the C-terminal end (93.73% identity). A third *MAT***a**2 coding sequence, namely *MAT***a**2 copy 3, encoded a protein 94.41% and 94.24% identical to ZrMAT**a**2 and ZsMAT**a**2, respectively.

The NJ-tree confirmed the high degree of evolutionary conservation of MAT**a**1 proteins within the *Z*. *rouxii* complex (bootstrapping 100%) (data not shown). Furthermore, a search with the program Pfam revealed that ATCC 42981 MAT**a**1 proteins conserve the HD2 class homeodomain [48] [49] (data not shown).

**Fig 3:**
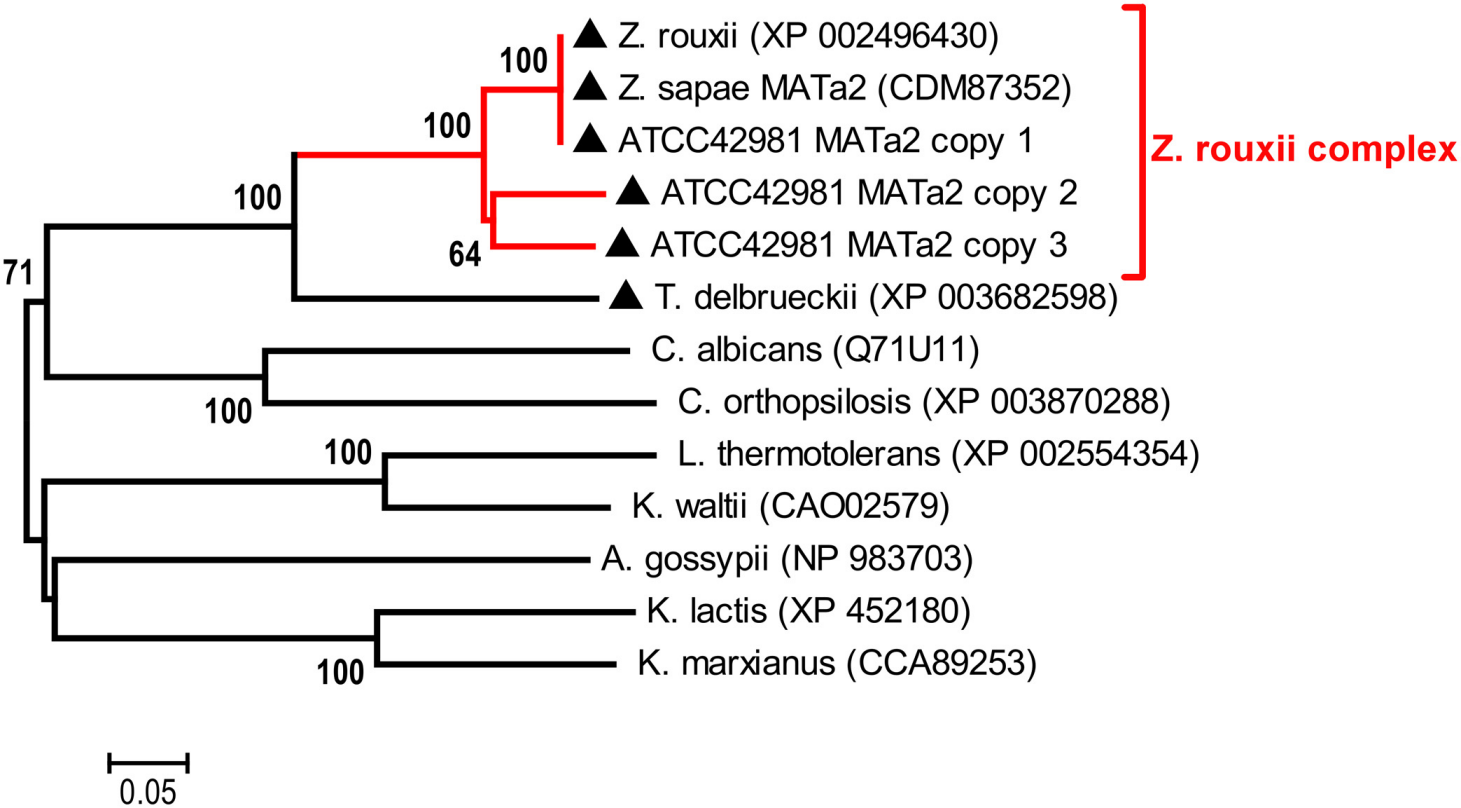
Phylogenetic analysis of MATa2 proteins. The neighbour-joining (NJ) tree shows the phylogenetic relationships between the allodiploid strain ATCC 42981 and other hemiascomycetes inferred from MAT**a**2 proteins. Numbers on branches indicate bootstrap support percentages (1,000 pseudoreplicates) higher than 60% from NJ. The red branch indicates the *Z*. *rouxii* complex, which includes *Z*. *rouxii* MAT**a**2, *Z*. *sapae* MAT**a**2 and ATCC 42981 MAT**a**2 copies 1 to 3 sequences. The dark dot indicates WGD species, whereas the dark triangle indicates non-WGD species with the *HO* gene.

**Fig 4:**
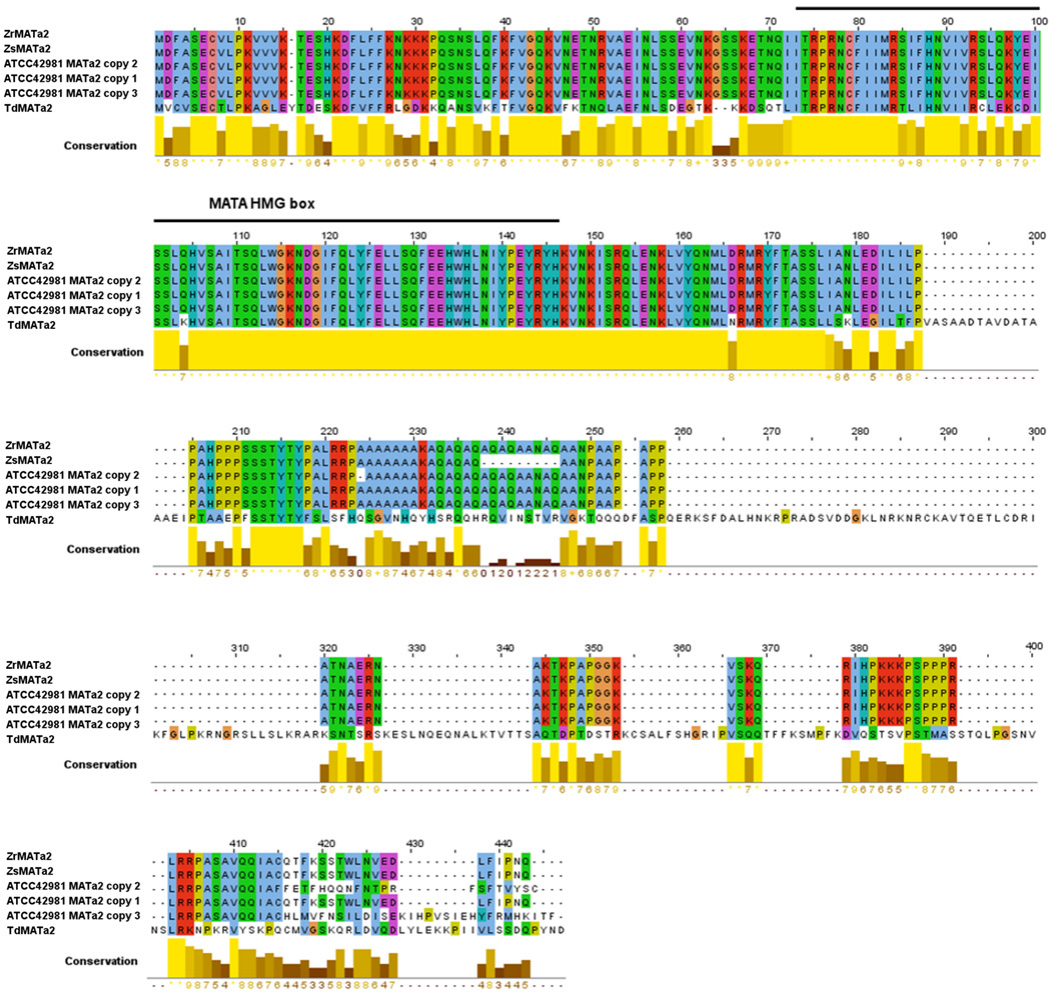
Sequence comparison of MATa2 proteins. Alignment of MAT**a**2 from *Z*. *rouxii* (ZrMAT**a**2, GenBank: XP002496430), *Z*. *sapae* (ZsMAT**a**2, GenBank: CDM87352), ATCC 42981 MAT**a** copies 1 to 3 and *Torulaspora delbrueckii* (TdMAT**a**2, GenBank: XP003682598). The MATA HMG domain, which binds the minor groove of DNA, is noted (horizontal black bar). In both alignments, the amino acid identities were coloured according the Clustal X colour scheme and the conservation index at each alignment position were provided by Jalview [50].

Phylogeny inferred from MAT**a**2 amino acid sequences showed that ATCC 42981 MAT**a**2 variants are phylogenetically distinct (Fig 3). In particular, MAT**a**2 copy 1 clustered with *Z*. *rouxii* and *Z*. *sapae* MAT**a**2 (bootstrapping 100%), whereas MAT**a**2 copies 2 and 3 clustered together and were distinct from both ZrMAT**a**2 and ZsMAT**a**2. MAT**a**2 proteins from ATCC 42981 conserved a single MATA-HMG box, a class I member of the HMG-box superfamily of DNA-binding proteins (Fig 4), coding a sequence spanning across the Y**a** and X regions.

### Characterization of *HO* genes

Using a PCR walking approach, we identified two 1797-bp full-length ORFs in the ATCC 42981 genome, termed *HO* copies 1 and 2, which diverged for 177 transitional and 49 transversional mismatches. The predicted proteins HO copies 1 and 2 shared 100% and 92.47% identity with *Z*. *rouxii* HO, while they were 100% and 99.83% similar to *Z*. *sapae* HO copies 1 and 2, respectively.

**Fig 5:**
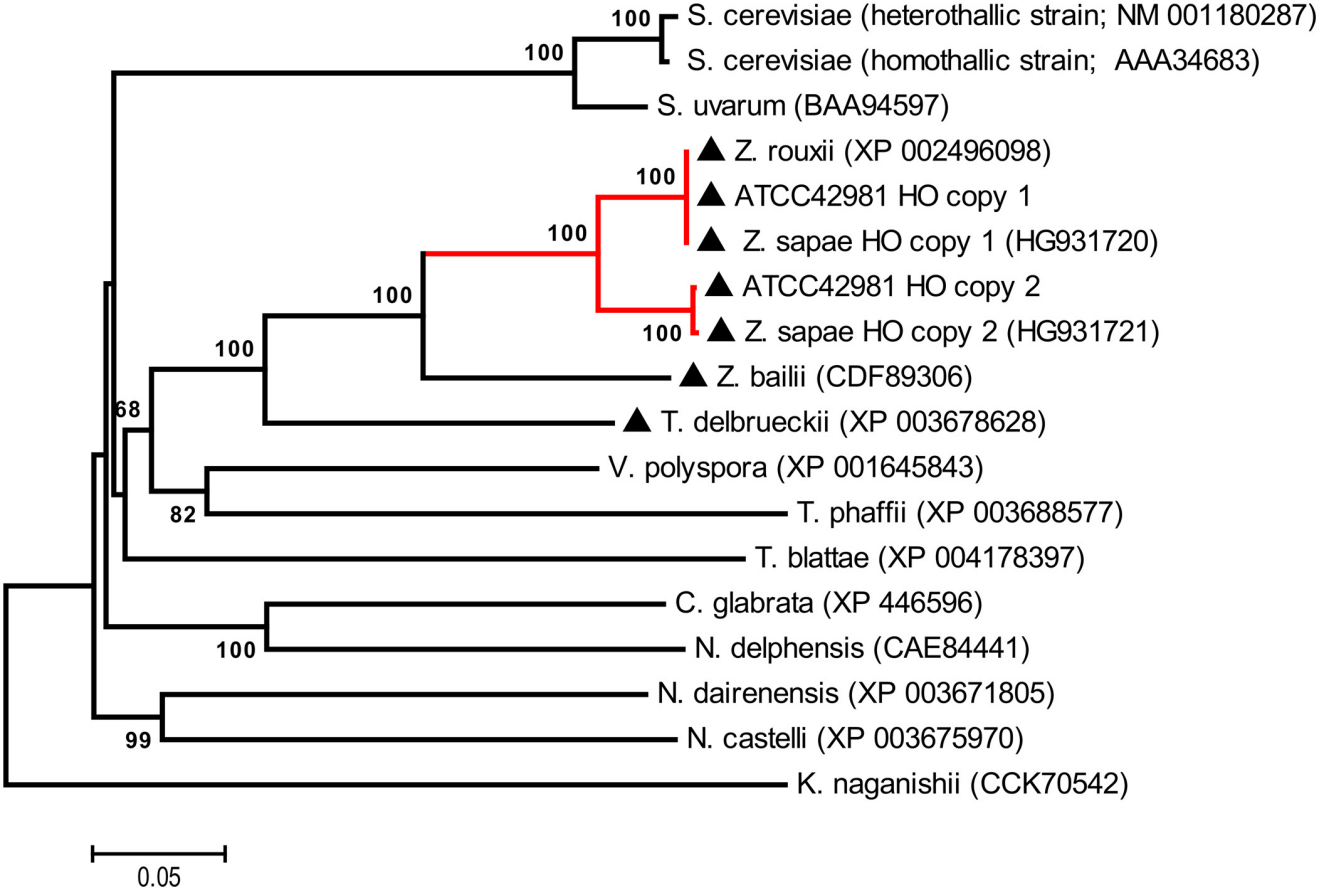
Phylogenetic analysis of HO endonucleases. Neighbor-joining (NJ) tree shows evolutionary relationships between ATCC 42981 strain and other hemiascomycetes as inferred from HO proteins. Numbers on branches indicate bootstrap support (1,000 pseudoreplicates) from NJ. The red branch indicates *Z*. *rouxii* complex, which includes ZrHO, ZsHOs and ATCC 42981 HO sequences, whereas the dark triangle designates pre-WGD species.

NJ-based phylogeny inferred from amino acid HO sequences confirmed that HO copy 2 from ATCC 42981 branched with *Z*. *sapae* HO copy 2 with a high level of support (bootstrapping 100%), while ATCC 42981 HO copy 1, ZsHO copy 1 and ZrHO clustered together into a separate clade (Fig 5). These results showed that ATCC 42981 HO amino acid sequences are more divergent from each other than from the putative orthologs in *Z*. *sapae*. Amino acid alignment of both ATCC 42981 HOs with *S*. *cerevisiae* PI-*Sce*I (GenBank: CAA98762), *S*. *cerevisiae* HO and *Z*. *sapae* HO copies 1 and 2 showed the highest homology in conserved motifs characteristic of intein-encoded LAGLIDADG endonucleases [51–53] (data not shown).

PFGE-Southern blotting showed that both *HO* copies of ATCC 42981 strain are on chromosome F (1.6 Mbp) (S4 Fig). This chromosomal assignment resembles that of CBS 732^T^, but differs from that previously found in *Z*. *sapae* ABT301^T^ [32]. We assumed that ATCC 42981 *HO* copies could be placed on the same chromosome or, alternatively, on two PFGE-co-migrating homeologous chromosomes.

### Subgenome assignment of mating-type and *HO* genes

In order to infer the putative parental contributions to the ATCC 42981 *MTL*α, *MAT***a** and *HO* gene sets, we screened strain NCYC 3042 for all variants of *MAT***a**, *MAT*α, and *HO* genes found in ATCC 42981. According to PCR profiles, successful amplification was obtained for copy 2 sets of *MAT*α1, *MAT*α2 and *HO* genes in the NCYC 3042 strain, while negative results were obtained for *MAT*α and *HO* copy 1 genes (S2 Table). PCR results were consistent with the high degree of similarity between the copy 1 set of *MAT*α and *HO* genes and *Z*. *rouxii* orthologs in the CBS 732^T^ genome project (Figs 1, 2 and 5) and suggested that *MAT*α and *HO* copies 2 originated from a *Z*. *pseudorouxii* parental genome. Interestingly, *MAT***a**2 copy-specific screening showed that the NCYC 3042 strain does not possess any *MAT***a**2 copies found in ATCC 42981 genome (S2 Table).

### Reconstruction of the three-cassette system

**Fig 6:**
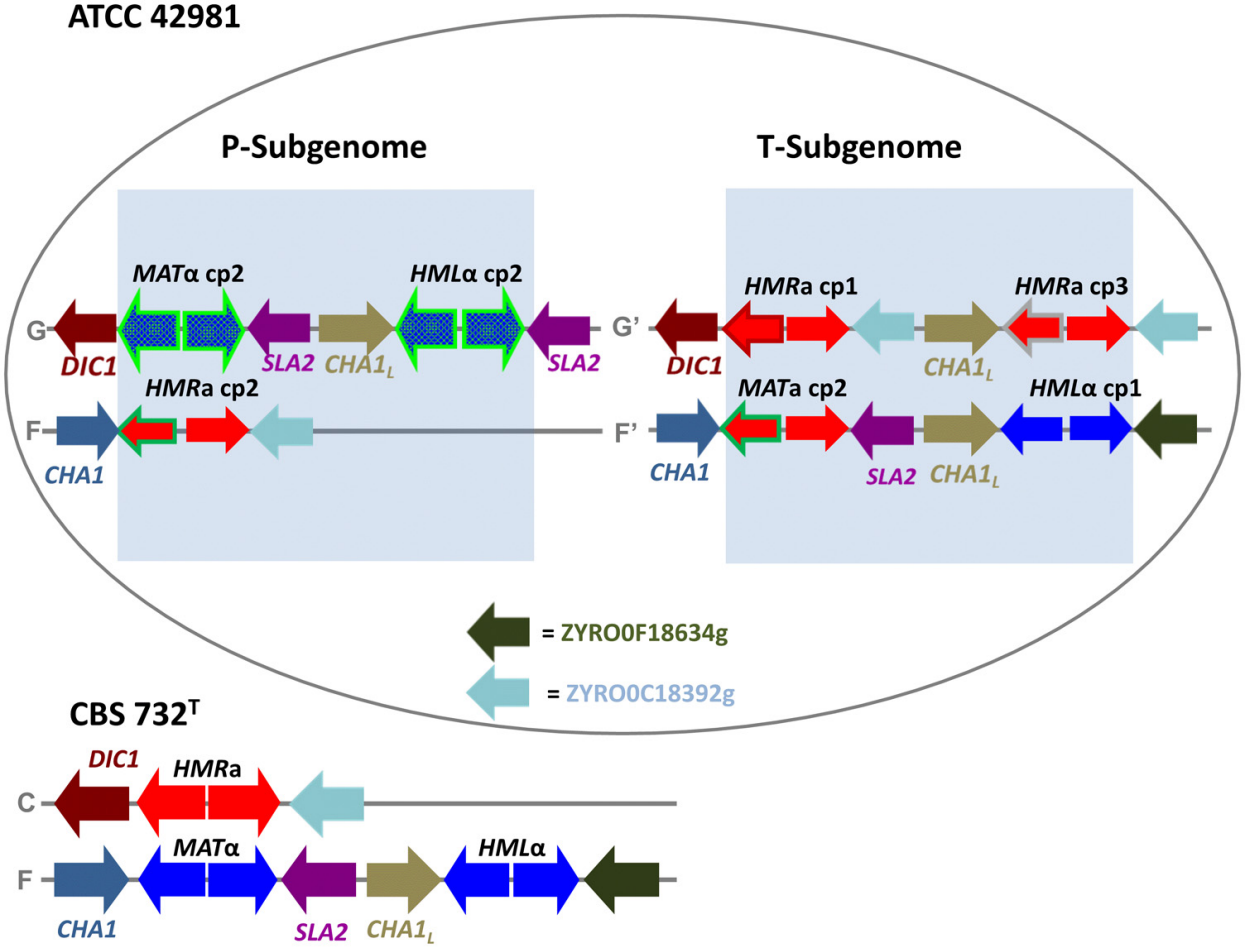
Inferred genomic organization around *MAT*-like loci in the ATCC 42981 allodiploid genome. Two sex homologous/homeologous chromosome pairs are depicted, namely F/F’ and G/G’. Chromosome G bears the *MAT*α copy 2 expression locus, which is linked to the putative silent cassettes *HML* copy 2, whereas chromosome F’ bears the *MAT***a** copy 2 expression locus, which is linked to the putative silent cassette *HML* copy 1. Chromosomes F and G’ harbour three **a**-idiomorph *HMR* loci, which differ for *MAT***a**2 genes. The *HMR* copy 2 locus is on chromosome F, while the *HMR* copies 1 and 3 loci are on chromosome G’. Blue arrows represent *Z*. *rouxii*-like (blue bordered) and *Z*. *sapae*-like (light green bordered) α-idiomorph loci. Red arrows indicate **a**-idiomorph loci with different *MAT***a**2 genes: copy 1 (dark red surrounded), copy 2 (dark green), copy 3 (grey), respectively. Chromosomal organization of the three-cassette system in *Z*. *rouxii* haploid strain CBS 732^T^ was reported for comparative purposes according to Souciet et al. [34]. *CHA1*_*L*_ indicates the ZYRO0F18524g locus, while dark green and light blue arrows indicate the ZYRO0F18634g and ZYRO0C18392g loci, respectively at the right side of mating-type loci.

To assign the *MTL***a** and *MTL*α loci as *MAT* expression loci, *HMR* or *HML* silent cassettes (Fig 6), we exploited direct PCR on *MTL* flanking regions and long-range PCRs that spanned left and right flanking regions of *MTL* loci (S2 Fig). Both approaches demonstrated that ZYRO0F18524g (termed *CHA1*_*L*_ due to its similarity to *CHA1* gene) and ZYRO0F18634g ORFs are located at the 5’ and 3’ ends of the *MTL*α copy 1 locus, respectively. Because *HML*α silent cassettes are commonly downstream the *CHA1*_*L*_ gene [31], we inferred the existence of a *HML*α silent cassette containing *MAT*α1 and *MAT*α2 copies 1 genes (referred to as *HML*α copy1) (Fig 6). Long-range PCR products obtained with the primer pairs 2/A and 3/A were positively screened through *MTL*α copy 2-specific primers (S2 Fig). Sequencing showed that there are two *MTL*α copy 2 loci flanked by the *SLA2* gene at the 3’ end and by either the *DIC1* gene or *CHA1*_*L*_ at the 5’ end, respectively. It has been reported that the *MAT*α expression loci retain the particular gene order *DIC1*-*MAT*α-*SLA2* in the majority of non-WGD species [54]. A similar synteny was found for *MTL*α copy 2, suggesting that ATCC 42981 possesses an actively transcribed *MAT*α copy 2 locus (Fig 6). Furthermore, *CHA1*_*L*_ upstream to the *MTL*α copy 2 supports the presence of an additional *HML*α copy 2 cassette. Finally, we detected the following syntenic orders for *MTL***a** loci: *DIC1*-*MTL***a** copy 1-ZYRO0C18392g, *CHA1*-*MTL***a** copy 2-*SLA2*, *CHA1*-*MTL***a** copy 2-ZYRO0C18392g and *CHA1*_*L*_-*MTL***a** copy 3-ZYRO0C18392g (Fig 6). The gene organization of the *CHA1*-*MTL***a** copy 2-*SLA2* was syntenic with that of the *MAT*α expression locus in *Z*. *rouxii* CBS 732^T^, whereas the gene orders *CHA1*-*MTL***a** copy 2-ZYRO0C18392g and *DIC1*-*MTL***a** copy 1-ZYRO0C18392g resembled those found in *HMR***a** silent loci of haploid *Z*. *rouxii* strains [31]. In particular, *CHA1*-*MTL***a** copy2-ZYRO0C18392g resembled the *HMR***a** cassette found in a- and α-idiomorph mixed culture of strain NBRC 1130. Although the synteny *CHA1*_*L*_-*MTL***a** copy 3-ZYRO0C18392g has been never found in *Z*. *rouxii* strains, *CHA1*_*L*_ and ZYRO0C18392g ORFs generally surround silent donor cassettes. Overall, these evidences support the notion that ATCC 42981 possesses one *MAT***a** copy 2 expression locus and three *HMR***a** cassettes, referred to as *HMR***a** copies 1 to 3 (Fig 6).

The chromosomal arrangement of *MAT*, *HMR* and *HML* cassettes was established by Southern blot analysis on PFGE-separated chromosome bands. Although PFGE-Southern blotting failed to clearly resolve the highest molecular weight chromosomes E, F and G spanning from 1.6 to 2.2 Mbp (S5 Fig), hybridization of PFGE-Southern blot with a *MAT*α1-specific probe (suitable to recognize both *MAT*α1 copies 1 and 2) resulted in a double band spanning from chromosome F to G for *Z*. *rouxii* CBS 732^T^ and strain ATCC 42981 (S5 Fig). However, differently from CBS 732^T^, in strain ATCC 42981, the *MAT*α1-specific probe bound less chromosome F than chromosome G, leading to two bands with different signal intensities. This result suggests that chromosome G harbours more copies of the *MTL*α cassette than chromosome F. When the same analysis was carried out with a *MAT***a**1-specific probe, we obtained a double band spanning from chromosome F and G of ATCC 42981 karyotype (S5 Fig). These studies suggest that *MTL***a** loci reside on at least two of the chromosomes spanning from 1.6 and 2.2 kbp. According to PFGE-Southern blotting results, the ATCC 42981 strain arranges two expressed *MAT* loci (*MAT***a** and *MAT*α copies 2), three *HMR***a** and two *HML*α silent cassettes on two homeologous chromosome pairs (G/G’ and F/F’). Taking into consideration that linked *MAT* and *HML* loci are located on a different chromosome compared to *HMR* [31] [54], we inferred the scenario depicted in Fig 6. Chromosome G putatively contains the expression locus *MAT*α copy 2, which is linked to the *HML*α copy 2 cassette, while chromosome F hosts the *HMR***a** copy 2 silent cassette. The expression locus *MAT***a** copy 2 is putatively located on chromosome F’, linked with *HML*α cassette copy 1. Chromosome G’ provides the two remaining silent *HMR***a** copies 2 and 3 cassettes (Fig 6).

### Gene expression analysis

**Fig 7:**
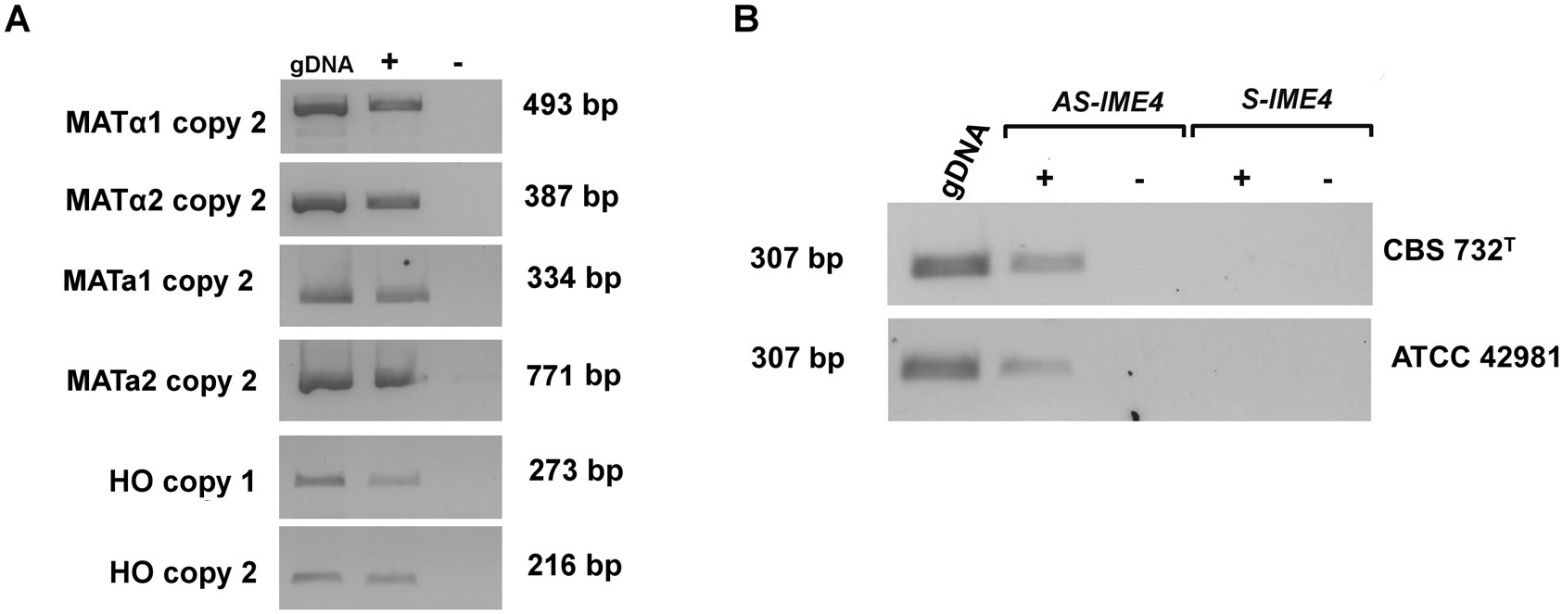
Expression pattern of mating-type, *HO* and *IME*4 genes. Panel A reports positive amplified cDNAs generated with *MAT* and *HO* copy variant-specific primers from ATCC 42981 unstressed cells in stationary growth phase. Panel B depicts *IME4*-specific PCR products generated from CBS 732^T^ and ATCC 42981 cDNA *IME4* antisense (AS-*IME4* lncRNA) and sense transcripts (S-*IME4* mRNA), respectively. +/- RT indicates addition of reverse transcriptase to the cDNA synthesis reaction. For each RT-PCR reaction gDNA was used as positive control. Abbreviations: AS, anti-sense long non-coding RNA; S, sense mRNA.

Non-quantitative RT-PCR confirmed that the expression loci *MAT*α and *MAT***a** copies 2 are actively transcribed in ATCC 42981 cells grown in standard conditions (Fig 7). Congruently to the cassette reconstruction, the *MAT*α1 and *MAT*α2 genes from *HML*α copy 1 were silent.

According to the regulatory circuit reported in *S*. *cerevisiae* diploid cells, we expected that h-sg sets are silenced by a functional **a**1-α2 heterodimer. In contrast, gene expression profile analysis revealed that the *MAT*α1 and *HO* genes were actively transcribed in the ATCC 42981 allodiploid strain. Similarly in haploid CBS 732^T^ unstressed cells *HO* gene was actively transcribed (data not shown).

To verify whether other members of the h-sg set were expressed, we tested the presence of S-*IME4* and AS-*IME4* transcripts. RT-PCR analysis showed that salt-stressed ATCC 42981 diploid cells only transcribed AS-*IME4*, while S-*IME4* was not detected (Fig 7). This pattern is similar to that displayed by haploid cells of the reference strain CBS 732^T^, which does not show any PCR products for S-*IME4* specific RT-PCR (data not shown). The identity of AS-*IME4* PCR products was confirmed by direct sequencing. We concluded that ATCC 42981 is a diploid strain that only transcribes the AS-*IME4* transcript.

### Transcriptional response to hyperosmotic stress

**Fig 8:**
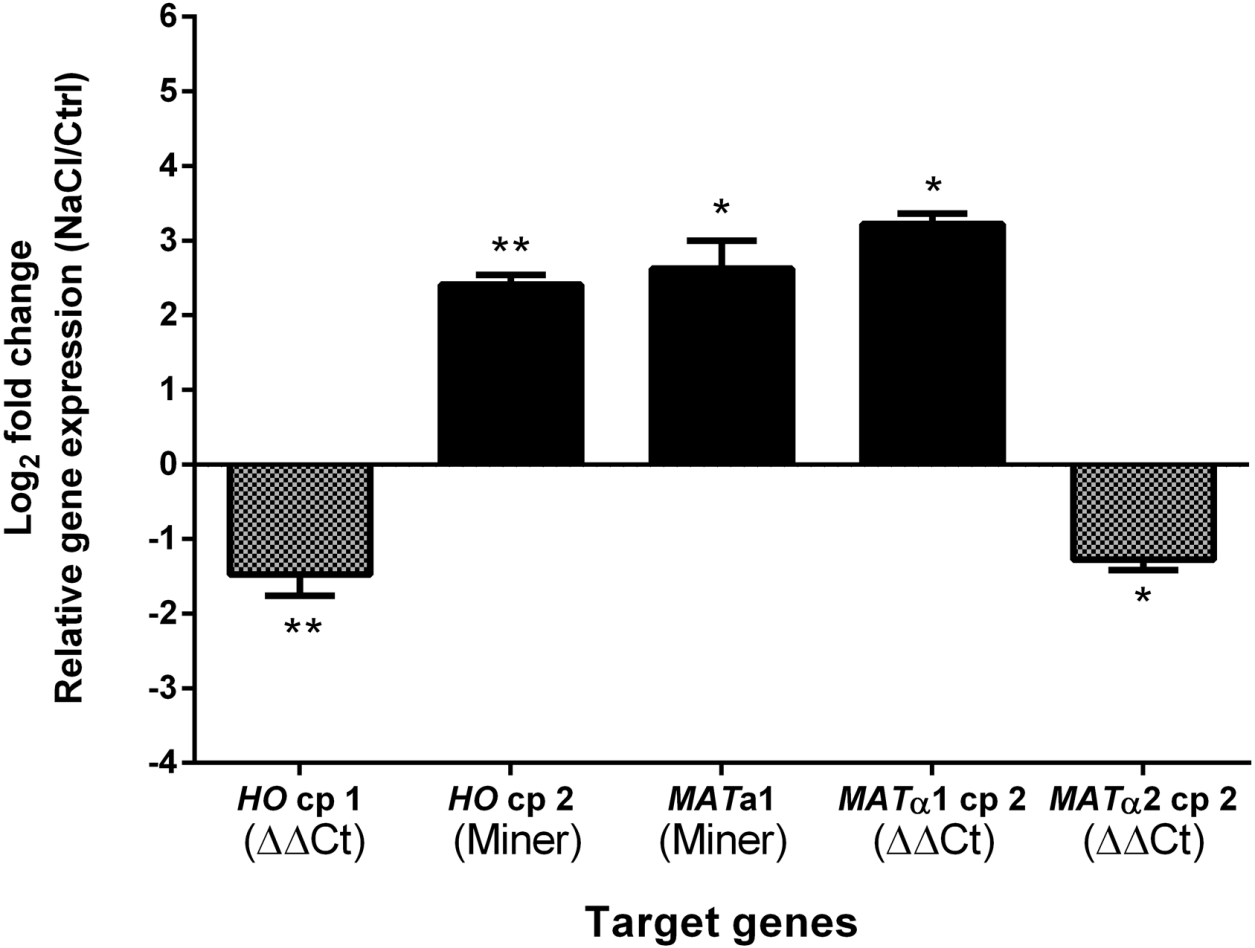
Differential expression by quantitative real-time PCR of mating-type and *HO* genes in ATCC 42981 cells after hyperosmotic stress. Expression of target genes was normalized on the reference *ZrACT1* (GenBank: XM002497273). Fold change was measured by ΔΔCt or PCR Miner methods and reported as the mean (± SEM) of three biological replicates. * indicates significant difference from controls as measured by independent Student's *t*-tests (*P<0.05, **P<0.01).

After hyperosmotic stress, ATCC 42981 cells exhibit a different expression profile for both mating-type and *HO* genes compared to controls. Salt stress induced higher transcript levels of *MAT***a**1 and *MAT*α1 copy 2 (6.2- and 9.3-fold, respectively). On the contrary, *MAT*α2 copy 2 expression was lower than that in controls (2.4-fold). *HO* copy 1 was slightly down-regulated (2.8-fold), whereas *HO* copy 2 was up-regulated (more than 5.3-fold) compared to controls (Fig 8).

## Discussion

Previous studies demonstrated that outcrossing is a very rare event (approximately once every 50,000/110,000 generations) in yeasts as *S*. *cerevisiae* [55], *Saccharomyces paradoxus* [56] and *Lachancea kluyveri* [57]. Nevertheless, it represents a potential source of phenotypic variability (novelty) and has been extensively exploited for the genetic improvement of microbial cell factories [58]. Although many inter-mating allopatric yeasts give rise to viable hybrids, post-zygotic isolating barriers [59] prevent gene flow between species and contribute to the process of speciation [60]. Complete sets of orthologous genes are expected in yeast hybrids immediately after the merging of two parental genomes. These patterns can undergo extensive homogenization processes over evolutionary time through intragenic recombination, gene conversion and differential gene loss, shaping the offspring’s genome architectures [61] [62]. Gordon and Wolfe [29] did not find any traces of gene losses in the ATCC 42981 strain, arguing that the allopolyploidization was so recent that its genome has not had enough time to decay. These authors hypothesized that one parental subgenome resembles *Z*. *rouxii* CBS 732^T^ and the other *Z*. *pseudororuxii* (nom. inval.) NCYC 3042. However, ATCC 42981 karyotype cannot be simply considered as an additive result between the putative parental counterparts, suggesting that some structural rearrangements have occurred in the ATCC 42981 genome compared to *Z*. *rouxii* and *Z*. *pseudorouxii* [28] [29]. Similarly, the *Z*. *rouxii* CBS 732^T^ strain contains a *SOD2-22* gene variant [63], while *Z*. *pseudorouxii* NCYC 3042 has *SOD2* [30]. The detection of *SOD2* and *SOD22* genes, but not the *SOD2*-*22* variant in the ATCC 42981 genome, further confirms that the subgenome complements are similar but not identical to those found in *Z*. *rouxii* CBS 732^T^ and in *Z*. *pseudorouxii* NCYC 3042.

In an allodiploid karyotype, two homeologous sex chromosomes are expected, bearing **a**- and α-idiomorph expression loci, respectively. Previous works demonstrated that in haploid *Z*. *rouxii* and diploid *Z*. *sapae*, *HMR* cassettes are located on a different chromosome compared to the *MAT*-*HML* linkage, accounting for the presence of two sex chromosomes [31] [32]. Therefore, in a recent allodiploid *Zygosaccharomyces* genome, we expected to identify two sex chromosome pairs. Accordingly, the ATCC 42981 strain displays an **a**/α genotype provided by the contribution of two sex chromosome pairs. The first sex chromosome pair consists of chromosomes G (*MAT*α copy 2-*HML*α copy 2) and F (*HMR***a** copy 2). This subgenome complement only partially resembles that found in *Z*. *pseudorouxii* NCYC 3042 strain [30] which might have contributed to this complement with *MTL*α copy 2. If *Z*. *rouxii* contributed to the second chromosome set, we would expect to detect a *Z*. *rouxii MAT***a**2 copy 1 gene at the expression locus. In contrast, chromosome F’ (carrying *MAT***a** copy 2-*HML*α copy 1) and G’ (carrying *HMR***a** copy 1-*HMR***a** copy 3) probably derived from a rearranged *Z*. *rouxii* genome complement [29], consistently with the mating-type loci being the hotspot of ectopic recombination in *Z*. *rouxii* strains [31]. Furthermore, an extra *HMR***a** copy 3 was detected harbouring another partially divergent *MAT***a**2 ORF, which it might have derived from another non-*Z*. *rouxii* parental strain or resulted from an ectopic recombination event in the allodiploid ancestor. All these findings are not consistent with a simple additive set of mating-type loci recently assembled in the allodiploid genome and indicate that ATCC 42981 T- subgenome differs from its putative *Z*. *rouxii* CBS 732^T^ counterpart. Watanabe et al. [31] found that the copy 2 variant of *MAT***a**2 gene is at the *HMR* silent cassette of strain NBRC1130 which undergone mating-type switching, whereas copy 1 variant occurs in *MAT* expression locus. This genome assortment is specular to that found in ATCC 42981 T-subgenome and hints that different *MAT***a**2 copies co-exist within *Z*. *rouxii* species, harboured by *MTL* loci flanked by different genes at 5’ end. We speculated that these copy variants could be relicts of past mating-type switching events. Interestingly, *Z*. *rouxii* possesses a hybrid regulation system targeting **a**-specific genes (**a**-sgs), which consists of **a**2-mediated activation and α2-mediated repression [64]. In haploid cells, MAT**a**2 proteins with diverging C-terminal portions could be functionally equivalent in mediating the activation of the **a**-sg program.

Beyond divergent mating-type loci, the ATCC 42981 strain has also been reported to possess two divergent *HO* genes. Orthologous genes coming from each of the parental species should appear as paralogs in standard analyses because they are homologous genes encoded by the same genome [65]. However, the degree of divergence identified between *HO* gene copies 1 and 2 excludes their origin as paralogs from a recent gene duplication event and hints that *HO* copy 2 originated from the *Z*. *pseudorouxii* parental genome. The pattern of neutral mutations detected in the endonuclease functional domains indicates that both *HO* genes have been exposed to the same selective pressure.

Gene regulatory networks, such as cell-type specification circuit, have been shown to evolve significantly over time [66]. When the divergent components of these circuits are forced to interact in a hybrid background, positive or antagonistic epistatic interactions may take place [67]. Among the mechanisms cited to explain the observed loss of hybrid fertility, the Bateson-Dobzhansky-Muller (BDM) model proposes that hybrid sterility results from the lack of interaction or the malfunctioning of interacting alleles derived from divergent genomes [3] [68–70]. However, in some cases, inter-specific protein assemblies have been reported to generate novelties in protein-protein networks untested by selection in hybrid species [71] [72]. The reconstruction of the sex chromosome architecture in the ATCC 42981 genome indicates that the transcriptional regulators encoded at the *MAT* expression loci are phylogenetically divergent. In diploid cells, **a**1-α2 heterodimer acts as a master regulator of the shift from mitosis to meiotic growth under appropriate meiosis-inducing stimuli, as well as a repressor of the h-sgs under standard growth conditions. H-sgs contain the *MAT*α1 gene, which the **a**1-α2 heterodimer should repress by binding the bidirectional promoter located between *MAT*α1 and *MAT*α2 ORFs [12]. In allodiploid ATCC 42981, the lack of MATα1 silencing could be due to: the lack of a1 and α2 proteins; the failure in the heterodimer formations; the inability of putative chimeric **a**1 and α2 subunits to positively interact with *cis*-regulatory promoter sequences. Similarly to ATCC 42981, *S*. *cerevisiae* diploids showing mutations in either **a**1 or α2 transcriptional factors fail to turn off *MAT*α1 and other h-sgs [73] [74]. Additionally, Strathern et al. [75] reported that a diploid mutant with a truncated *MAT*α2 defective allele displays a weak α phenotype and is unable to sporulate. Overall, these evidences suggest that in ATCC 42981 the cell-type specification circuit is ineffective in repressing the *MAT*α1 gene possibly due to, among other factors, a no functional chimeric **a**1-α2 heterodimer.

In diploids, the entry into meiosis requires the inhibition of lncRNA AS-*IME4* by a functional **a**1-α2 heterodimer [21]. Conversely, our results show that the ATCC 42981 strain produces anti-sense transcripts for the *IME4* gene, leading to clonality as its unique mode of reproduction. The haploid-like transcriptional pattern displayed by stressed ATCC 42981 cells could also account for its increase of adhesive phenotype. In haploids grown under stress cues, a complex network of signalling modules and transcriptional factors induce clamp formation and pseudohyphal growth in order to enhance mating efficiency and chance of survival [76]. The *FLO11* gene has been reported to be a key determinant of the adhesion phenotype under the positive control of the RME1 transcriptional factor. In diploids, the *RME1* gene is silenced by a functional **a**1-α2 heterodimer, leading to the entry into meiosis in response to environmental cues. Because we observed more clamps in salt-stressed compared to unstressed ATCC 42981 cells, we argue that the chimeric **a**1-α2 heterodimer is also partially ineffective in repressing *RME1* transcription.

In *S*. *cerevisiae*, a redundant regulatory network accounts for the three-level regulation of mating-type interconversion catalysed by the *HO* endonuclease, namely cell-type control (*HO* gene is expressed in **a** or α haploid cells); mother-daughter control (*HO* is transcribed in the mother but not in the daughter cells); and cell-cycle control (*HO* is expressed during the late G1 phase of the cell cycle after the point of commitment to the next cell cycle) [77]. Three transcriptional repressors are involved: the **a**1-α2 heterodimer, *SIN3* and *SIN6*, respectively. These three types of negative constraints must be relieved in order for the *HO* gene to be transcribed. Allodiploid ATCC 42981 cells express *HO* mRNAs, probably due to the lack of these types of controls or to partial incompatibilities among transcriptional factors. However, the presence of *HO* transcripts does not imply that ATCC 42981 undergoes mating-type interconversion. Indeed, the transcriptional analysis of *MAT* expression loci showed that only the expected *MAT***a** copy 2 and *MAT*α copy 2 transcripts are detected in salt-stressed cells at the stationary phase. The failure of mating-type switching induced by salt stimuli could be due to either a *HO* post-transcriptional control or to the lack of a fully functional network controlling the DSB-initiated gene conversion. However, other effectors could be responsible for this event, as in haploid *Z*. *rouxii* strains *HO* deletion determines only a slight decrease in mating-type switching frequency [31]. The *Z*. *rouxii* species complex emerged after the ancestor of hemiascomycetous yeasts had diverged from other families, such as *Kluyveromyces*. *K*. *lactis* has a non-functional copy of the *HO* gene [78] and performs mating-type switching by an alternative transposase-mediated mechanism [79]. As it is the first non-WGD clade with a functional *HO* gene, the *Z*. *rouxii* complex is likely to retain remnants of both mechanisms.

In haploid *Z*. *rouxii*, mating and the subsequent zygote formation occur immediately before sporulation mainly under salt stress [24] [80]. Therefore, we investigated how the ATCC 42981 cells modulate the transcription of genes coding the **a**1 and α2 subunits, as well as that of their downstream h-sg targets *MAT*α1 and *HO* in response to long-term hypersaline stress. In diploid cells, the silencing of h-sg *MAT*α1 and *HO* and the positive regulation of the *MAT*α2 gene are controlled by a working **a**1-α2 heterodimer. The inability of the ATCC 42981 chimeric **a**1-α2 heterodimer to bind to the h-sg promoter regions may account for the observed up-regulation of *MAT*α1 and *HO* genes. Functional defects of the chimeric **a**1-α2 heterodimer could be due to gene incompatibility between two divergent subunits and/or to the transcriptional imbalance of their encoding genes. ATCC 42981 cells could attempt to overcome these functional deficiencies by over-expressing the components of the **a**1-α2 transcriptional factor. Congruently, we observed an up-regulation of the **a**1 transcript. A similar up-regulation of the other heterodimer subunit α2 should be expected. In contrast, *MAT*α2 gene expression appears to be down-regulated, even at a small level, suggesting an imbalance in the co-regulation of **a**1 and α2 subunits.

In conclusion, we demonstrated that allodiploid ATCC 42981 cells display a *MAT***a**/*MAT*α genotype with a chimeric sex-determination system originating from the co-existence of two different parental genome complements. The protein-protein interaction incompatibility between divergent **a**1 and α2 subunits could switch-off the meiosis commitment genes, contributing to ATCC 42981 allodiploid sterility. The presence of a chimeric **a**1-α2 heterodimer promotes an unusual haploid-like transcriptional profile in cells recovered at the stationary phase and after exposure to meiosis-inducing stimuli. To the best of our knowledge, this is the first cue that the BDM interaction between the divergent **a**1 and α2 subunits may act as a bottleneck, preventing genetic exchanges among *Zygosaccharomyces* species. Recently, a novel scenario has been proposed for yeast evolution, where two ancient non-WGD ancestral species have given rise to an allodiploid cell that has doubled its genome in order to restore fertility (with a possible interval of many mitotic generations between these two events) [81]. Interestingly, one of the possible parents exhibits phylogenetic affinities with the non-WGD *Z*. *rouxii* clade. However, there are several critical open questions that still need to be answered, such as: how the genome duplication event took place and how the mechanism of restoring fertility operated [65]. Allodiploid ATCC 42981 and other strains belonging to the *Zygosaccharomyces* mosaic lineage [23] could serve as promising models to shed light on the transcriptional network incompatibility underlying hybrid sterility at an incipient stage of speciation, and more in general, on yeast genome evolution.

## Supporting Information

S1 Fig Outlined experimental strategy used for characterizing *MTL*α (A) and *MTL*a loci (B) in the ATCC 42981 genome. Panel A shows *ZsMTL*α variants represented in blue and surrounded in grey (copy 1), green (copy 2) and orange (copy 3), respectively, while panel B represents *MTL***a** loci coloured in red. Primer pairs specific for *ZsMTL*α copies 1 to 3 are arbitrarily referred to as αCp1-P, αCp2-P, and αCp3-P. *ZsMAT***a**1 and *ZsMAT***a**2-specific primer pairs are arbitrarily referred to as P**a**1 and P**a**2, while the primer pair termed P**a**12 spans the complete *MAT***a**1 ORF and a portion of *MAT***a**2 gene. Solid grey arrows indicate generic flanking genes, dotted borders represent uncompleted sequences and small arrows (solid) designate gene-specific primers. Primer sequences are reported in S1 Table, according to [31] and [32]. Abbreviations: Zs, *Zygosaccharomyces sapae*; cp, copy; P, primer; for, forward; rev, reverse.
(TIF)

S2 Fig Polymerase chain reaction (PCR)-based strategies used for determining the system of cassette-based arrangement in the ATCC 42981 genome. Forward and reverse *MTL*-specific internal primers were used to screen PCR products obtained using all possible combinations of primers spanning putative *MTL*-flanking genes (semi-nested PCR approach); in cases of negative results, 5' and 3' PCR walking was performed using all possible combinations of *MTL*-specific internal primers and *MTL*-flanking gene primers (direct PCR approach). Small arrows (solid) indicate gene-specific primers and degenerate primers (dotted lines). *CHA1*_*L*_ indicates the ZYRO0F18524g locus. Primer sequences are reported in S1 Table, according to [31] and [32]. Abbreviations: cp, copy; for, forward; rev, reverse.
(TIF)

S3 Fig Outlined experimental approach used for *HO* gene characterization in the ATCC 42981 genome. Dotted lines represent undetermined sequences. Primer sequences are reported in S1 Table. Abbreviation: *ZsHO*, *Zygosaccharomyces sapae HO* gene.
(TIF)

S4 Fig Chromosomal mapping of ATCC 42981 *HO* genes. Chromosomes were separated by PFGE for ATCC 42981 (1), *Z*. *rouxii* CBS 732^T^ (2), and *Z*. *sapae* ABT301^T^ (3). Southern blotting analysis was carried out with probe labelled to *HO* genes. M indicates the chromosomal size ladder (*Saccharomyces cerevisiae* S288C, BioRad Laboratories) in megabase pairs. ATCC 42981 chromosomes are indicated in uppercase letters (from A to G).
(TIF)

S5 Fig Chromosomal mapping of ATCC 42981 *MTL*α and *MTL*a loci. Chromosomes were separated by PFGE for *Z*. *rouxii* CBS 732^T^ (1) and ATCC 42981 (2). Southern blotting analyses were carried out with probes labelling the α- and **a**-idiomorph loci. The left panel shows signals from *MTL*α loci *Zygosaccharomyces rouxii* CBS 732^T^ (1) and the ATCC 42981 strain (2), respectively. The right panel reports separated chromosomes and signals from *MTL***a** loci in ATCC 42981 (2). M indicates the chromosomal size ladder (*Saccharomyces cerevisiae* S288C, BioRad Laboratories) in megabase pairs. ATCC 42981 chromosomes are indicated in uppercase letters (from A to G).
(TIF)

S1 Table List of primers used in the present study. (XLS)

S2 Table PCR-based subgenome assignment of mating-type and *HO* gene copies in *Zygosaccharomyces pseudorouxii* (nom. inval.) NCYC 3042. (DOCX)
