# Supplementary figures and images for: Chimeric Sex-Determining Chromosomal Regions and Dysregulation of Cell-Type Identity in a Sterile Zygosaccharomyces Allodiploid Yeast
Source: PLoS One. 2016 Apr 11;11(4):e0152558. doi: 10.1371/journal.pone.0152558 (PMC4827841; doi:10.1371/journal.pone.0152558)

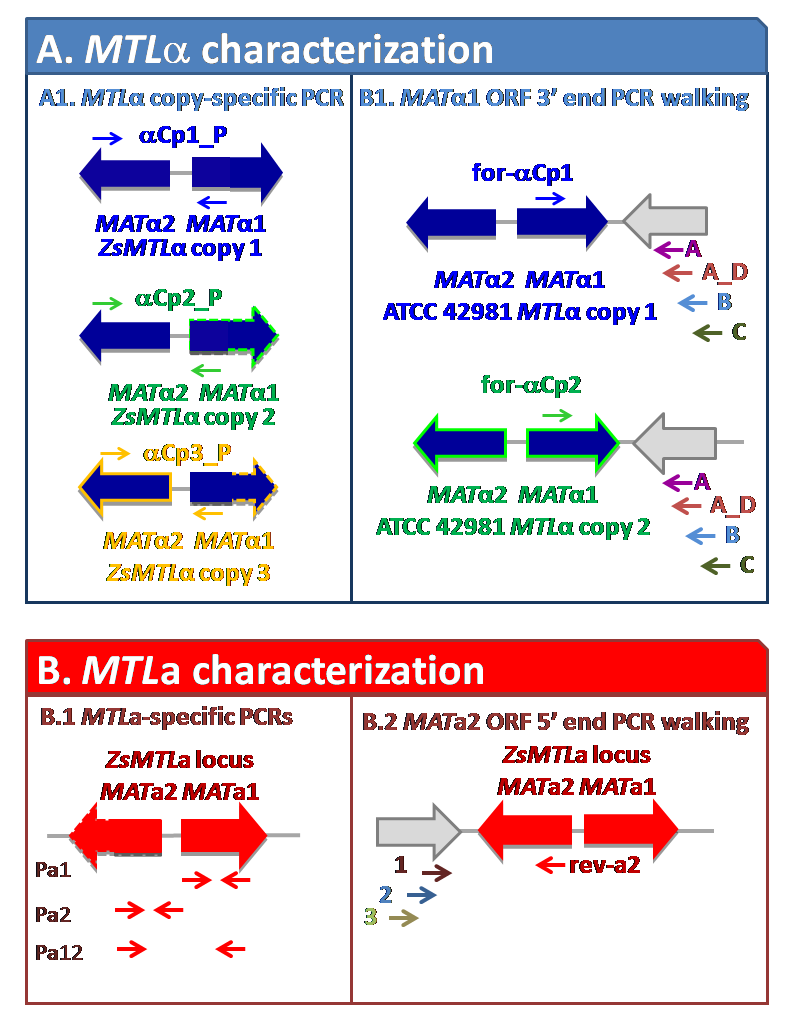

Supplement: S1 Fig — Panel A shows ZsMTLα variants represented in blue and surrounded in grey (copy 1), green (copy 2) and orange (copy 3), respectively, while panel B represents MTLa loci coloured in red. Primer pairs specific for ZsMTLα copies 1 to 3 are arbitrarily referred to as αCp1-P, αCp2-P, and αCp3-P. ZsMATa1 and ZsMATa2-specific primer pairs are arbitrarily referred to as Pa1 and Pa2, while the primer pair termed Pa12 spans the complete MATa1 ORF and a portion of MATa2 gene. Solid grey arrows indicate generic flanking genes, dotted borders represent uncompleted sequences and small arrows (solid) designate gene-specific primers. Primer sequences are reported in S1 Table, according to [31] and [32]. Abbreviations: Zs, Zygosaccharomyces sapae; cp, copy; P, primer; for, forward; rev, reverse. (TIF) [file pone.0152558.s001.tif]

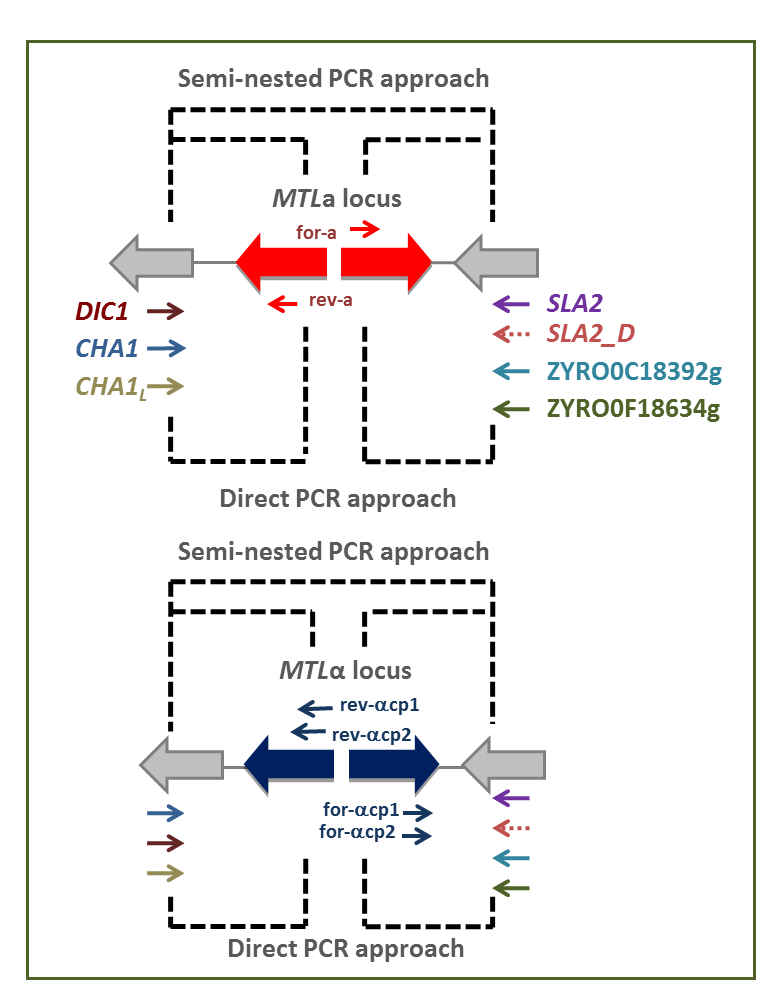

Supplement: S2 Fig — Forward and reverse MTL-specific internal primers were used to screen PCR products obtained using all possible combinations of primers spanning putative MTL-flanking genes (semi-nested PCR approach); in cases of negative results, 5' and 3' PCR walking was performed using all possible combinations of MTL-specific internal primers and MTL-flanking gene primers (direct PCR approach). Small arrows (solid) indicate gene-specific primers and degenerate primers (dotted lines). CHA1L indicates the ZYRO0F18524g locus. Primer sequences are reported in S1 Table, according to [31] and [32]. Abbreviations: cp, copy; for, forward; rev, reverse. (TIF) [file pone.0152558.s002.tif]

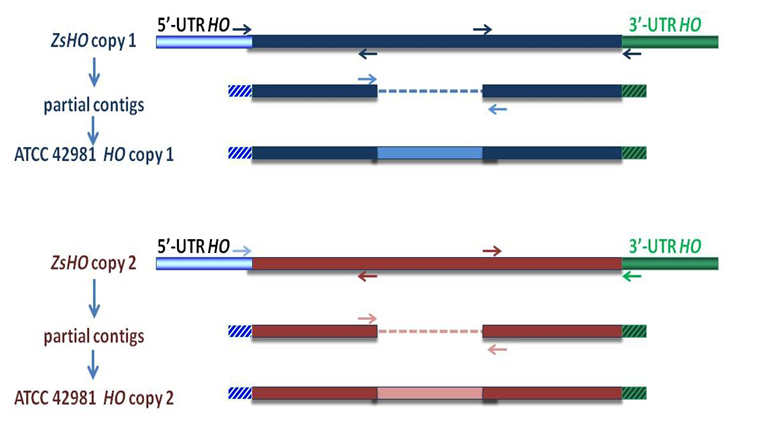

Supplement: S3 Fig — Dotted lines represent undetermined sequences. Primer sequences are reported in S1 Table. Abbreviation: ZsHO, Zygosaccharomyces sapae HO gene. (TIF) [file pone.0152558.s003.tif]

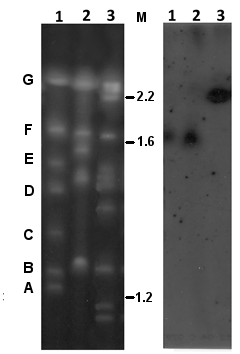

Supplement: S4 Fig — Chromosomes were separated by PFGE for ATCC 42981 (1), Z. rouxii CBS 732T (2), and Z. sapae ABT301T (3). Southern blotting analysis was carried out with probe labelled to HO genes. M indicates the chromosomal size ladder (Saccharomyces cerevisiae S288C, BioRad Laboratories) in megabase pairs. ATCC 42981 chromosomes are indicated in uppercase letters (from A to G). (TIF) [file pone.0152558.s004.tif]

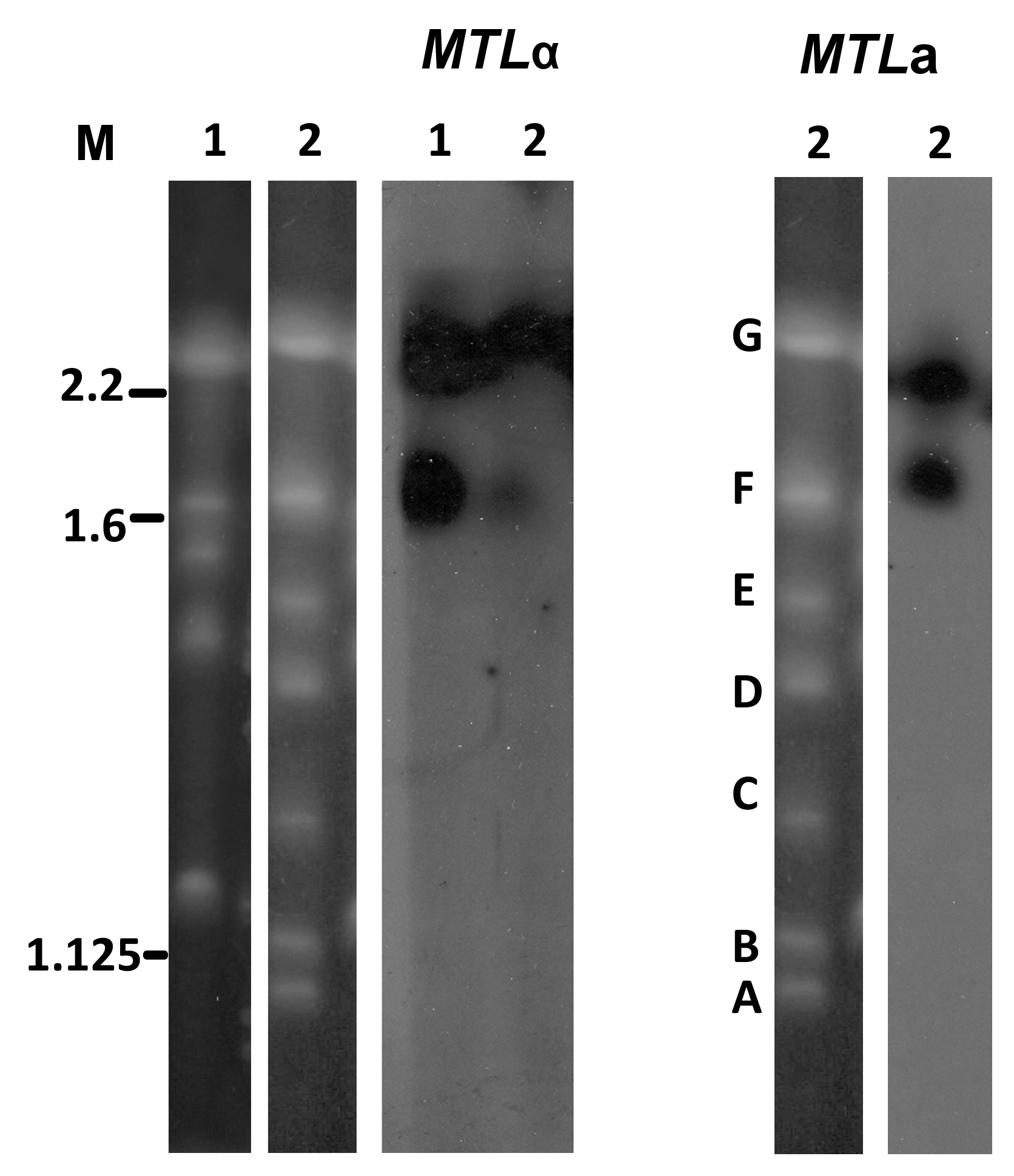

Supplement: S5 Fig — Chromosomes were separated by PFGE for Z. rouxii CBS 732T (1) and ATCC 42981 (2). Southern blotting analyses were carried out with probes labelling the α- and a-idiomorph loci. The left panel shows signals from MTLα loci Zygosaccharomyces rouxii CBS 732T (1) and the ATCC 42981 strain (2), respectively. The right panel reports separated chromosomes and signals from MTLa loci in ATCC 42981 (2). M indicates the chromosomal size ladder (Saccharomyces cerevisiae S288C, BioRad Laboratories) in megabase pairs. ATCC 42981 chromosomes are indicated in uppercase letters (from A to G). (TIF) [file pone.0152558.s005.tif]
