# Supplementary material for: Chimeric Sex-Determining Chromosomal Regions and Dysregulation of Cell-Type Identity in a Sterile Zygosaccharomyces Allodiploid Yeast
Source: PLoS One. 2016 Apr 11;11(4):e0152558. doi: 10.1371/journal.pone.0152558 (PMC4827841; doi:10.1371/journal.pone.0152558)
Supplement: S2 Table — (DOCX) [file pone.0152558.s007.docx]

**S2 Table. PCR-based sub-genome assignment of mating-type and *HO* gene copies in *Zygosaccharomyces pseudorouxii* (nom. inval.) NCYC 3042.**

| **Target gene** | **NCYC 3042** | | |
| --- | --- | --- | --- |
|  | **copy 1** | **copy 2** | **copy 3** |
| ***MAT*α1** | - | + | nd |
| ***MAT*α2** | - | + | nd |
| ***MAT*a1** | - | - | - |
| ***MAT*a2** | - | - | - |
| ***HO*** | - | + | na |

nd, not determined; na, not applicable; +, positive result; -, negative result
